# Supplementary material for: Evaluating the Memory Enhancing Effects of Angelica gigas in Mouse Models of Mild Cognitive Impairments
Source: Nutrients. 2019 Dec 30;12(1):97. doi: 10.3390/nu12010097 (PMC7019643; doi:10.3390/nu12010097)
Supplement: Supplementary file 1 [file nutrients-12-00097-s001.pdf]

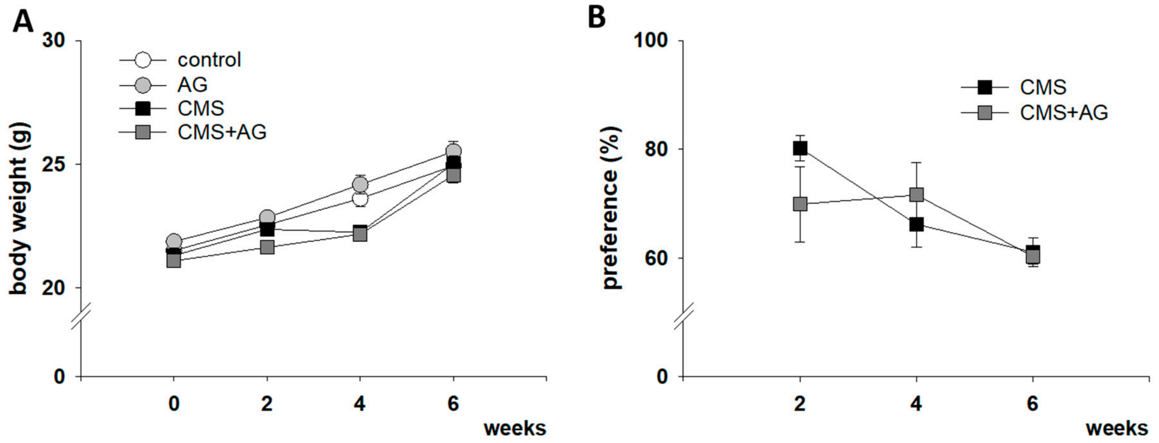

Figure S1. Body weight and sucrose preference change in CMS mice

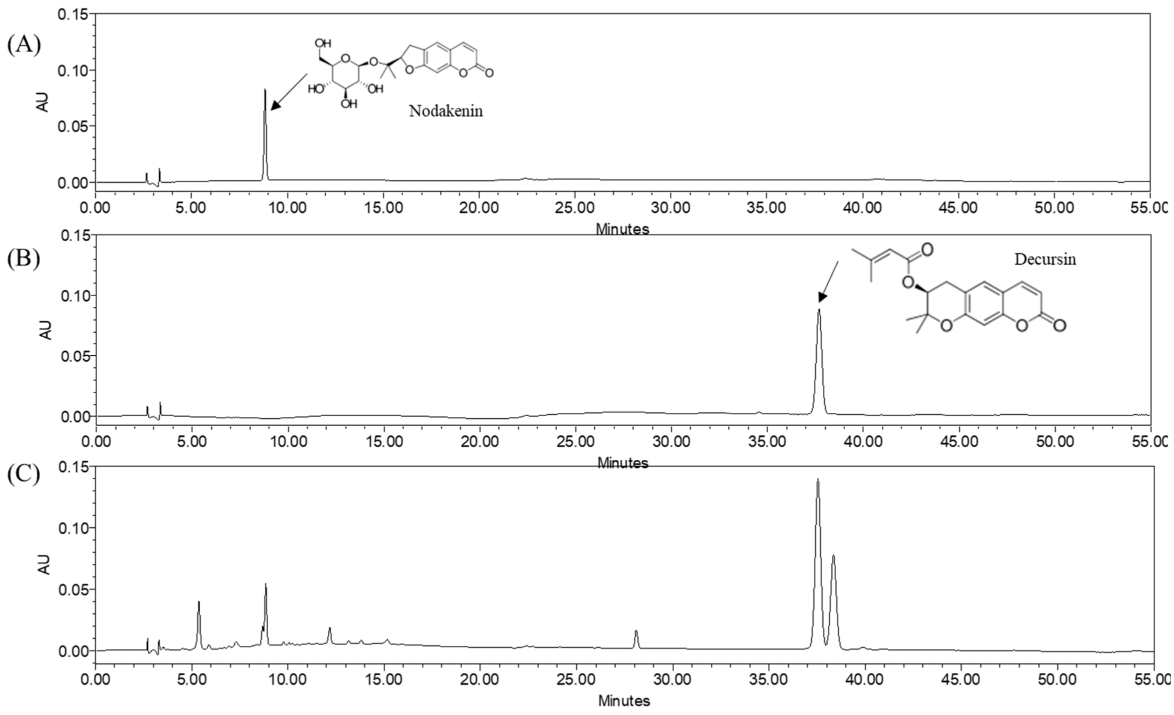

Figure S2. HPLC analysis of AG extract. The HPLC analysis was performed on a Waters liquid chromatograph with photodiode array detector interfaced with an Empower 2 for the data analysis. The column was a 4.6 mm ID×250 mm CAPCELL PAK C18 column (5 μm pore size). The mobile phase was composed of water (a) and acetonitrile (b) using the following linear gradient program: 0~3 min, 20% ; 3~8 min, 20~30% ; 8~18 min, 30% ; 18~19 min, 30~50 % ; 19~40 min, 50% ; 40~43 min, 50~90% ; 43~50 min, 90% ; 50~55 min, 90~20% B. Chromatography was carried out in gradient mode using a flow rate of 1.0 ml/min at 30℃ and was detected at various UV wavelength of individual standards and AG sample. Two standards (Nodakenin and Decursin) were prepared at 20 ug/mL and 262.5 μg/ml, respectively. AG extract powder was dissolved in 50%MeOH at 1 mg/ml. (A) The retention time of Nodakenin was 8.2 minute measured at 335 nm. (B) The retention time of Decursin was 37.7 minute measured at 325 nm. (C) HPLC chromatogram of AG sample was measured at 325 nm.
